# Screening-testing approaches for gene-gene and gene-environment interactions using independent statistics

**DOI:** 10.3389/fgene.2013.00306

**Published:** 2013-12-30

**Authors:** Joshua Millstein

**Affiliations:** Division of Biostatistics, Department of Preventive Medicine, Keck School of Medicine, University of Southern CaliforniaLos Angeles, CA, USA

**Keywords:** epistasis, multiple testing, case-only, two-step, two-stage, filtering, screening

Next-generation sequencing and other high-throughput technologies have made it feasible to characterize millions of sequence variations on large numbers of study participants. But when it comes to identifying a small number of these genetic features (or feature sets) that are associated with a disease trait, the investigator is faced with a formidable multiple-testing challenge. It can be thought of as a signal-to-noise problem, where the large number of unrelated genetic features tends to drown out the faint signal of the small number of biologically relevant features.

The theoretical underpinnings of an emerging class of statistical methods for genomic studies, two-stage procedures for both gene-gene and gene-environment interactions have recently been described in a remarkable article (Dai et al., 2012). The key idea is that dimensionality of multiple testing in genomics can be reduced by screening features to be tested with an independent statistic in the same dataset, thereby mitigating the multiple-testing problem and increasing power to detect effects. In other words, the noise is reduced, allowing the relevant signal to be more easily detected. These methods will likely gain importance as high-throughput technologies continue to yield exponentially increasing amounts of information per sample and per research dollar spent.

Dai et al. couched their paper in the context of gene-environment interactions only. However, it is worth noting that the theoretical properties detailed by Dai et al. apply not just to the search for gene-environment interactions (GxE), but also to (epistatic) interactions between genetic variants (GxG), since in constructing these hypothesis tests, both “gene” and “environment” features are treated analogously as discrete or continuous variables in models designed to identify associations with a disease trait. A notable exception is when the approach depends on the environmental exposure being a randomized treatment, allowing additional assumptions to be made.

One such screening-testing interaction approach is designed for a case-control study where the investigator is interested in identifying GxG or GxE pairs involved in interactions (Millstein et al., 2006; Murcray et al., 2009; Dai et al., 2012; Lewinger et al., 2013). There is an assumption that each pair of features considered is independent in the general population, and only if a dependence is found in the pooled case-control sample (the screening stage), is the pair tested in a formal model that includes an interaction term (the testing stage), e.g., logit(*P*[*D*]) = α + β^*^_1_SNP1 + β^*^_2_SNP2 + β^*^_3_SNP1^*^SNP2, where β_3_ is the interaction parameter and *D* indicates disease. The interaction parameter can be tested alone or in a multi degree-of-freedom test of one or both main effects together with the interaction, an approach that was generally found to be more powerful (Millstein et al., 2006; Kraft et al., 2007). An important characteristic of the approach is that even if the independence assumption is not justified, type I error in the testing stage will still be properly controlled.

This approach is perhaps more general and more powerful than previously appreciated. The screening procedure appears to be sensitive to both main effects and interactions, not just interactions, as claimed in prior work. The implication is that the approach is less specific to interactions and correspondingly more powerful when main effects are present. In fact, it may be capable of detecting weak interactions coupled with weak main effects. Some authors (Murcray et al., 2009; Dai et al., 2012; Lewinger et al., 2013) have attributed the statistical power of the screening procedure solely to an association in cases due to an interaction in the underlying population (non-zero β_3_, or more correctly, a departure from multiplicativity on a *relative risk* scale), as in the case-only interaction analysis (Piegorsch et al., 1994). According to this view, controls only contribute noise to the screening procedure because the factors are independent in this population. Further, if the two features contribute marginal disease risks and a multiplicative relative risk model describes their joint risk, then dependencies will not be induced among cases. The idea is that if there is independence in cases and independence in controls, then it should follow that there would be independence in the pooled case-control sample—but this is not necessarily the case. It has not been adequately appreciated that when cases and controls are pooled, main effects can contribute a substantial increase in power to capture disease-related feature pairs with the above screening procedure. Interestingly, the complex conditioning on disease status inherent in pooling of cases and controls can induce dependencies and thus increase power of the screening procedure when main effects are present.

**Figure 1:**
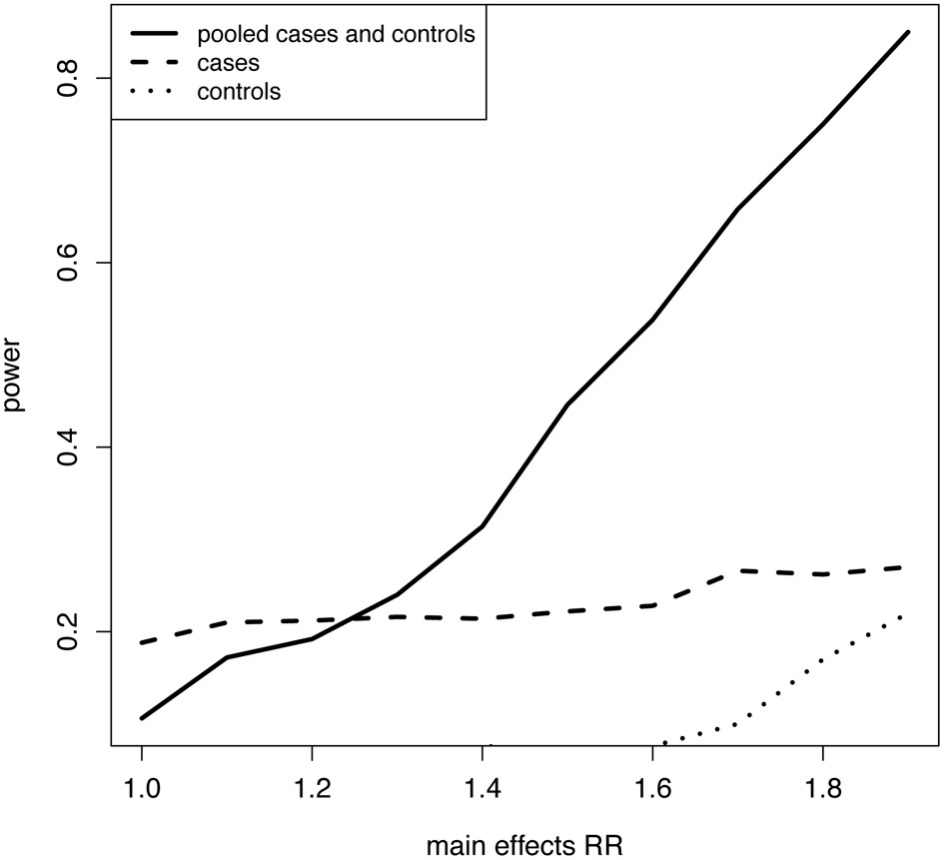
**Power in cases, controls, and a pooled case-control sample for a screening procedure based on LD between SNPs.** Empirical power calculations were plotted for detection of composite LD between two SNPs, independent in the general population, coded additively in the number of minor alleles, with minor allele frequencies of 0.2. Detection of composite LD was performed by applying the t-distribution to Pearson's product moment correlation coefficient with α = 0.05. A population of 40000 individuals including a binary disease trait was simulated according to the model, log(*P*[*D*]) = λ + β^*^_1_SNP1 + β^*^_2_SNP2 + β^*^_3_SNP1^*^SNP2, where baseline risk of disease was 5%, main effects were equal, β_1_ = β_2_, and the interaction was weak exp(β_3_) = 1.1. Power of the screening procedure as described above is plotted over a range of main effect relative risks [main effects RR = exp(β_1_) = exp(β_2_)] for a sample of 1000 cases only, 1000 controls only, and a pooled case-control sample of 1000 cases and 1000 controls randomly sampled from the population.

As proof of concept, consider the relatively simple relative risk model, log(*P*[*D*]) = λ + β^*^_1_SNP1 + β^*^_2_SNP2 + β^*^_3_SNP1^*^SNP2, where exp (λ) is the baseline risk, the two SNPs have equal relative risks per allele, i.e., β_1_ = β_2_, there is a weak interaction (small β_3_), and equal numbers of cases and controls sampled for a moderately rare disease. It is apparent that power to identify the disease related SNPs using a screening approach based on composite LD in the pooled case-control sample can increase with the strength of the main effects and be quite powerful despite a weak interaction (Figure 1). This result may explain why using the pooled cases and controls generated a more effective screening tool than cases only, in an application to identify SNPs that modify estrogen treatment efficacy in the Woman's Health Initiative study (Dai et al., 2012). It is up to future studies to clarify just how much and under what conditions main effects and controls contribute to the power of the approach.

Dai et al. made a major contribution to screening-testing designs by providing rigorous proofs that (1) screening-testing approaches are valid if independent statistics are used for the screening and testing stages, and (2) various pairs of statistics are independent. However, there is general way to conceptualize some of these approaches that leads to an intuitive understanding of conditions that preserve type I error, thus allowing investigators to develop novel and complex screening-testing approaches without having to develop new proofs. In the types of studies under discussion, the ultimate objective is often to detect some form of association between the genetic features and the disease trait, conditional on the ascertainment scheme, that is, to find evidence of associations in the study sample that reflect dependencies in the general population. Thus, the corresponding null hypotheses consist of some form of independence between features of interest and the disease trait both in the underlying population and conditional on ascertainment. If we define the global null hypothesis to be independence between the feature vector and the disease trait, then any function of the features is also independent of the disease trait under the null, due to basic properties of independence of random variables (Cassella and Berger, 2002). Therefore, if a screening statistic is strictly a function of the genetic variables and does not depend on disease status, then the global null is equally valid for the reduced set of variables, and only the reduced set of tests needs to be considered for multiple testing correction. Millstein et al. (2006) used this rational to justify the screening-testing approach described above. Also, Millstein and Gauderman (2002) proposed a related screening-testing approach for identifying multi-SNP interactions in case-control studies that involved estimation of an analytically intractable parameter. The screening statistic was based on the density of the most dense multi-locus genotype clusters in the pooled case-control sample. However, it was intuitively clear to the authors (and demonstrated empirically) that proper control of type I error was achieved by the approach, because the screening procedure did not depend on disease status in any way (aside from being conditional on ascertainment).

Screening-testing interaction approaches have the potential to be applied across a broad array of study designs, and have already branched out beyond the original case-control application. For instance, there have been applications for studies of nuclear families (Millstein et al., 2005) and trios (Gauderman et al., 2010) as well as survival analysis (Dai et al., 2012). But there are many other potential applications that have not yet been proposed. For instance, there are many studies that are designed to investigate traits within a diseased population, such as response to treatment, age of onset, or disease progression. It is easy to envision a scenario where features that interact to confer risk of disease also interact to affect disease traits. It would seem that a powerful screening-testing approach for this scenario would be analogous to the approach described above, that is, identify feature pairs with evidence of unexpected dependencies (screening), and then test them for joint effects on disease traits (testing). Proper type I error control in the testing stage is preserved under the null of independence between the features and the disease traits. For example, if we are conducting a study of obese patients, we could perform the screening procedure using all of these patients, looking for dependencies between features (G-G or G-E) that we expect to be independent in the general population, effectively conducting a case-only interaction analysis but without requiring statistical significance. Then in the testing stage the top feature pairs from the screening procedure would be tested for joint effects on BMI as a continuous outcome in a linear model using data from the same obese study participants. The Bonferroni correction would only need to account for the number of tests that were conducted in the testing stage.

Screening-testing interaction approaches could have even broader applications if we are able to relax the assumption of independence between the screening and testing statistics. The methods could then be applied to other designs, such as cross-sectional and cohort studies. For example, suppose we think that some pair of features may jointly affect disease, having both main effects and interactions. We may want to screen on the interaction effect but jointly test main effects and interactions in the testing stage, since joint tests can be much more powerful (Millstein et al., 2006; Kraft et al., 2007) and to avoid missing an important discovery if in truth there is a strong interaction but weak or non-existent main effects. Clearly, in this case we would not have independence between the screening and testing statistics. However, GWAS analyses are often conducted under the assumption of exchangeability of observations under the null. And under the assumption of exchangeability, one can often construct a permutation procedure to control family-wise type I error or estimate FDR, even when the distribution of the test statistic is not known or not accurate. Such an approach may seem computationally infeasible for a genome-wide application, but very fast epistasis screening procedures have recently been developed (Kam-Thong et al., 2011), and it is demonstrated elsewhere in this issue that accurate estimates of FDR can be generated with as few as 10 permutations (Millstein and Volfson, 2013). Thus, under the assumptions of exchangeability and independence between the features and the disease trait under the null, the above screening-testing approach or related approaches could be applied and FDR estimated, even when relatively few permutations are conducted. The Millstein and Volfson approach includes a confidence interval estimator for FDR that accounts for the number of permutations conducted, thereby quantifying uncertainty, which is especially useful in the presence of weak effects and small numbers of permutations.

Given the breadth of previously proposed applications as well as potential new directions and insights discussed here, it seems likely that the use of screening-testing interaction approaches will prove to have a big impact on future identification of multi-locus as well as GxE effects.
